# Contribution of apoptosis-associated signaling pathways to epileptogenesis: lessons from Bcl-2 family knockouts

**DOI:** 10.3389/fncel.2013.00110

**Published:** 2013-07-16

**Authors:** David C. Henshall, Tobias Engel

**Affiliations:** Department of Physiology and Medical Physics, Royal College of Surgeons in Ireland, St. Stephen's GreenDublin, Ireland

**Keywords:** apoptosis, bcl-2, brain, hippocampus, mitochondria, neuron, stroke, temporal lobe epilepsy

## Abstract

Neuronal cell death is a pathophysiological consequence of many brain insults that trigger epilepsy and has been implicated as a causal factor in epileptogenesis. Seizure-induced neuronal death features excitotoxic necrosis and apoptosis-associated signaling pathways, including activation of multiple members of the Bcl-2 gene family. The availability of mice in which individual Bcl-2 family members have been deleted has provided the means to determine whether they have causal roles in neuronal death and epileptogenesis *in vivo*. Studies show that multiple members of the Bcl-2 family are activated following status epilepticus and the seizure and damage phenotypes of eight different knockouts of the Bcl-2 family have now been characterized. Loss of certain pro-apoptotic members, including Puma, protected against seizure-induced neuronal death whereas loss of anti-apoptotic Mcl-1 and Bcl-w enhanced hippocampal damage. Notably, loss of two putatively pro-apoptotic members, Bak and Bmf, resulted in more seizure-damage while deletion of Bid had no effect, indicating the role of certain Bcl-2 family proteins in epileptic brain injury is distinct from their contributions following other stressors or in non-CNS tissue. Notably, Puma-deficient mice develop fewer spontaneous seizures after status epilepticus suggesting neuroprotection may preserve functional inhibition, either directly by preserving neuronal networks or indirectly, for example by limiting reactive gliosis and pro-inflammatory responses to neuronal death. Together, these studies support apoptosis-associated molecular mechanisms controlling neuronal death as a component of epileptogenesis which might be targetable to protect against seizure-damage, cognitive deficits and mitigate the severity of syndrome following epilepsy-precipitating injuries to the brain.

## Introduction

Epileptogenesis is the process by which a normal brain is transformed into one capable of generating recurrent spontaneous seizures (Pitkanen and Lukasiuk, 2011; Goldberg and Coulter, 2013). The process can be triggered by injuries to the brain such as trauma, stroke, infection and acute symptomatic seizures such as status epilepticus. Interrupting this process would offer the possibility of preventing the development of epilepsy, a chronic and often life-long neurologic disorder characterized by an enduring predisposition to seizures (Chang and Lowenstein, 2003). If complete prevention is not possible, a secondary goal would be to modify the severity of the condition, for example resulting in fewer or less severe seizures (e.g., partial seizures rather than generalized tonic-clonic) (Pitkanen and Lukasiuk, 2011).

Current anti-epileptic drugs (AEDs) do not appear to significantly modify the underlying pathophysiology, with the possible exception of levetiracetam and ethosuximide in immature models (Loscher and Brandt, 2010; Pitkanen and Lukasiuk, 2011). This implies that the mechanisms of ictogenesis and epileptogenesis are not the same (Sloviter and Bumanglag, 2013). This underscores the need to understand the cell and molecular mechanisms underlying epileptogenesis and then identify potentially novel approaches and targets with which to design anti-epileptogenic treatments.

There has been major progress in characterizing the cell and molecular processes that occur during epileptogenesis [for review, see Mcnamara et al. (2006); Boison (2008); Engel and Henshall (2009); Pitkanen and Lukasiuk (2011); Vezzani et al. (2011); Goldberg and Coulter (2013)]. Epileptogenesis is associated with acute and delayed neuronal death, gliosis (particularly astrocytes and microglia), changes to synaptic and circuit function (morphology, channelopathies, firing properties, γ-amino butyric acid (GABA)-ergic tone), neuroinflammation, aberrant neurogenesis and extracellular matrix remodeling. We remain uncertain which individual processes or combination of processes are important and some undoubtedly reflect repair processes that are beneficial (Loscher and Brandt, 2010; Sloviter, 2011). Efforts to target epileptogenesis and prevent the subsequent emergence of epilepsy have been largely unsuccessful to date (Loscher and Brandt, 2010; Pitkanen and Lukasiuk, 2011).

## Neuronal death as a consequence of seizures and causal factor in epileptogenesis

Neuron loss within the hippocampus is a common pathologic hallmark of mesial temporal lobe epilepsy (mTLE) in humans. The most frequent finding is end folium sclerosis (loss of neurons within the hilus) and loss of CA1 and CA3 pyramidal neurons. Area CA2 and dentate granule neurons are usually spared, although some neurons may also be lost from these regions (Chang and Lowenstein, 2003; Thom, 2009). The neuron loss in mTLE is mainly thought to be a result of an initial precipitating insult such as prolonged febrile seizures, infection, trauma or status epilepticus. Prolonged or repeated epileptic seizures, however, may contribute to further neuron loss (Mathern et al., 2002; Henshall and Meldrum, 2012).

Animal models of mTLE produced by repeated brief seizures or status epilepticus are also associated with significant neuron loss within the hippocampus, as well as various other pathologic changes including gliosis, inflammation, blood brain barrier damage and circuit reorganization. How might neuronal death contribute to epileptogenesis or shape the epileptic phenotype? Both direct and indirect mechanisms are possible. Neuron loss may cause an imbalance between excitation and inhibition with brain networks, for example by removing inhibitory neurons or by removing excitatory neurons that function to activate inhibitory neurons (Pitkanen, 2002; Loscher and Brandt, 2010; Sloviter, 2011). The immediacy of epilepsy in some models supports a direct pro-epileptogenic effect of neuronal death. For example, spontaneous seizures are detected with minimal delay after intense hippocampal activation via perforant path stimulation that destroys neurons in the dentate hilus (Bumanglag and Sloviter, 2008). CA3-restricted lesions of the hippocampus also produce epilepsy with minimal latency suggesting neuronal death in this region is also epileptogenic (Li et al., 2008; Mouri et al., 2008). Neuron loss is also implicated as an epileptogenic mechanism following injury to thalamo-cortical circuitry (Paz et al., 2010). Epilepsy has also been reported to develop with no significant latent period following status epilepticus-induced brain injury in humans (Mikaeloff et al., 2006). Other supporting evidence includes the strong association found in some studies between epileptic seizure rates and the extent of damage to the neocortex (Kharatishvili and Pitkanen, 2010) and hippocampus (Jimenez-Mateos et al., 2010). Neuronal death may have pro-epileptogenic effects through indirect mechanisms; reactive gliosis and inflammation accompany neuronal damage and can promote hyperexcitability (Maroso et al., 2010; Vezzani et al., 2011). Recent transcriptome analysis of epileptogenesis identified genes regulating apoptosis as common to the epileptogenic process (Okamoto et al., 2010) and mice lacking the apoptosis-regulating gene *Chop* exhibit increased hippocampal damage and cognitive deficits following status epilepticus and develop more frequent spontaneous seizures (Engel et al., 2013).

If neuronal death is important for epileptogenesis and/or shaping the emergent phenotype then neuroprotection should have anti-epileptogenic effects. There are, however, relatively few studies which have provided direct evidence. A common problem is that drugs have been given before or during status epilepticus and this has modified the initial precipitating injury (Acharya et al., 2008; Loscher and Brandt, 2010; Sloviter, 2011). Excluding such studies, we are left with some examples where neuroprotection was found to be anti-epileptogenic. Injection of rats with a neuroprotective dose of a caspase-3 inhibitor 3 h after status epilepticus strongly reduced the number of spontaneous seizures and the proportion of rats developing epilepsy (Narkilahti et al., 2003). Neuroprotection through seizure preconditioning is also associated with reduced spontaneous seizures (Jimenez-Mateos et al., 2008). Neuroprotection also protects against cognitive deficits such as memory impairment as well as behavioral changes (Loscher and Brandt, 2010).

Not all studies, however, have found a convincing link between neuronal death and epileptogenesis. For example, glutamate receptor antagonist and valproate treatment after status epilepticus protected against cell death but did not prevent epileptogenesis (Acharya et al., 2008; Loscher and Brandt, 2010). Genetic deletion of p53 was recently shown to result in a more severe epilepsy phenotype despite animals displaying smaller hippocampal lesions after status epilepticus (Engel et al., 2010d). This may reflect possible “neuro-overprotection” or circumstances where loss of neurons, particular irrevocably damaged cells, aids the tissue repair process and leads to improved functional recovery (Andre et al., 2000; Gilby et al., 2005). Last, the role of neuronal death may be less important in the developing brain where epileptogenesis has been reported without overt cell death (Raol et al., 2003; Dube et al., 2006).

## Genetic tools to address the role of neuronal death in epileptogenesis

Pharmacological neuroprotection is rarely complete in status epilepticus models, leaving open the question of whether full protection, if feasible, would have a stronger anti-epileptogenic effect. Pharmacological tools also have limitations, including the question of when to deliver, how much, for how long and the duration of action (Sloviter, 2011). We also lack specific drugs to many of the pathways implicated in the control of neuronal death. Genetic models enable an assessment to be made based on the complete absence of a particular protein during the injury. Such tools also enable us to understand the causal roles of the genes in the signaling pathways that coordinate cell death.

**Figure 1:**
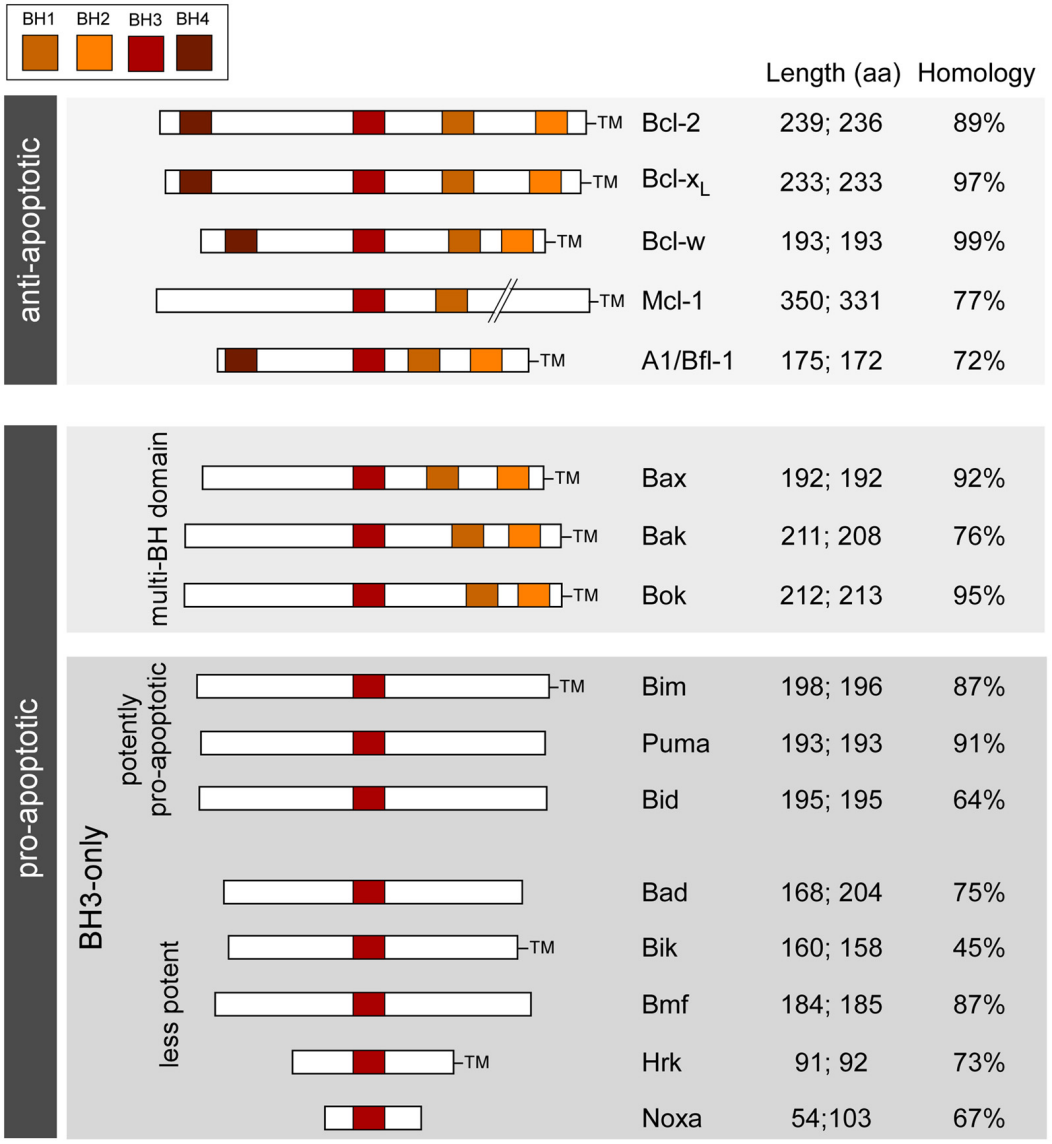
**Bcl-2 family; organization, functions, and characteristics.** Schematic shows the main Bcl-2 family proteins in humans and mice along with the main conserved structural motifs (BH domains), grouped according to function and depicting amino acid length and homology between species. TM, transmembrane domain. Length refers to amino acid number in (left side) *homo sapiens* and (right side) *mus musculus* (splice variants not included). Homology is between human and mouse amino acid sequences (BLAST).

Which genes to target? Genes involved in apoptosis-associated signaling are attractive because (1) apoptosis-associated signaling is a molecular feature of seizure-induced neuronal death; (2) extensive mouse lines have been developed to study the role of these genes in other diseases (mainly cancer), including targeting entire families; and (3) the genes are highly conserved between mice and humans (Figure 1). The Bcl-2 family represents a large group of genes for which multiple knockout mice exist and is the focus of the present article. In the past decade, researchers have characterized seizures and the damage phenotype in mice lacking eight different members of the family. Together, the findings reveal a select role for members of the Bcl-2 family in seizure-induced neuronal death and evidence that cell death controlled by Bcl-2 family proteins is functionally important in epileptogenesis (Table 1). Reviews on the broader topic of apoptosis and epilepsy can be found elsewhere [see Henshall and Simon (2005); Engel and Henshall (2009); Bozzi et al. (2011)].

**Table 1:** **Summary of effects of genetic disruption of Bcl-2 family proteins on status epilepticus-induced neuronal death and post-status epilepsy in mice**.

**Gene**	**Expected role**	**SE model**	**Chemoconvulsant/SE threshold in knockout**	**Hippocampal pathology in knockout**	**Effect on post-SE epilepsy**	**References**
*Mcl-1^a^*	Anti-apoptotic	Pilocarpine	Lower threshold for convulsions	Increased CA3 damage	Not tested	Mori et al., 2004
*Bcl-w*	Anti-apoptotic	i.a. KA	Earlier onset of SE	Increased CA3 damage	Not tested	Murphy et al., 2007
*Bak*	Pro-apoptotic	Systemic KA	Increased seizures^b^	Increased CA3 damage	Not tested	Fannjiang et al., 2003
		PTZ	Same as wt			
*Bim*	Pro-apoptotic	i.h. KA	Same as wt	Same as wt	Not tested	Theofilas et al., 2009
		i.a. KA	Same as wt	Reduced CA3 damage	Not tested	Murphy et al., 2010
		Systemic KA	Same as wt	Not reported	Not tested	Gimenez-Cassina et al., 2012
*Bid*	Pro-apoptotic	i.a. KA	Same as wt	Same as wt	Not tested	Engel et al., 2010a
		Systemic KA	Same as wt	Not reported	Not tested	Gimenez-Cassina et al., 2012
*Puma*	Pro-apoptotic	i.a. KA	Same as wt	Reduced CA3 damage	Reduced epileptic seizures	Engel et al., 2010c
		i.a. KA (high)^c^	Same as wt	Reduced CA3 damage	Not tested	Engel et al., 2010b
*Bad*	Pro-apoptotic	Systemic KA, PTZ	Reduced seizures	Not reported	Not tested	Gimenez-Cassina et al., 2012
*Bmf*	Pro-apoptotic	i.a. KA	Same as wt	Increased CA3, CA1 and hilar damage	Increased epileptic seizures^d^	Moran et al., 2013

a Studies performed using heterozygous, not knockout mice.

b Behavioral assessment, not by EEG.

c Higher dose (1 μg) of KA was used to produce more severe SE.

d Mice displayed ~30% more seizures although effect did not reach statistical significance.

Abbreviations: KA, kainic acid; i.a., intra-amygdala; i.h., intra-hippocampal; PTZ, pentylenetetrazole; SE, status epilepticus; SzPC, seizure preconditioning; wt, wild-type.

## Bcl-2 family proteins

Bcl-2 family proteins are the gatekeepers of the intrinsic (mitochondrial) pathway of apoptosis (Youle and Strasser, 2008; Hotchkiss et al., 2009; Chipuk et al., 2010). The intrinsic pathway of apoptosis is triggered by intracellular disturbances such as DNA damage, hypoxia, calcium overload, growth factor withdrawal, oxidative stress and misfolded proteins (Galluzzi et al., 2009; Tait and Green, 2010). Mitochondria are central to the convergence of these signals, and in the molecular processes which result in either restitution of cell homeostasis or execution of cell death. Loss of mitochondrial membrane potential constitutes a critical, irreversible step in cell death with subsequent release of pro-apoptogenic proteins (cytochrome c, apoptosis inducing factor), bioenergetic failure and downstream activation of caspase-dependent and caspase-independent cell death (Hotchkiss et al., 2009).

The Bcl-2 family are pivotal to the initiation, integration and execution of the intrinsic apoptosis pathway. About twenty Bcl-2 family members are recognized in mammals, based on sequence and function and they are typically organized into three groups (Figure 1). The anti-apoptotic members include Bcl-2, Bcl-xL, Bcl-w, and Mcl-1. Each member shares four Bcl-2 homology (BH) domains and a transmembrane domain that anchors them to intracellular membranes, particularly to mitochondria (Chipuk et al., 2010). Pro-apoptotic members are divided into the multi-BH domain “effectors” and the BH3-only proteins. The effectors comprise Bax and Bak. They share BH1-3 domains and have a transmembrane domain. Bok, a third putative member of this sub-group based on structural similarities, appears to be less important and cannot promote apoptosis in the absence of Bax/Bak (Echeverry et al., 2013). Activation of Bax/Bak involves oligomerization and pore formation in mitochondrial membranes that triggers mitochondrial outer membrane permeabilization and release of apoptogenic proteins (Galluzzi et al., 2009; Chipuk et al., 2010).

BH3-only proteins are a highly heterogeneous group of structurally unrelated proteins with the exception that they share a BH3 domain in common (see Figure 1). The most accepted model is that BH3-only proteins function either as “sensitizers,” including Bad, or “direct activators,” such as Bim and Bid. Sensitizers bind and neutralize anti-apoptotic Bcl-2 family proteins lowering the threshold for apoptosis, but not directly causing mitochondrial outer membrane permeabilization or apoptosis. Direct activators are capable of interacting with multi-domain Bax/Bak and triggering their insertion into mitochondria (Galluzzi et al., 2009; Chipuk et al., 2010).

## Seizures activate multiple Bcl-2 family proteins

Multiple members of the Bcl-2 family have been found to undergo transcriptional and/or post-translational responses following status epilepticus in rat and mouse models. Seizures cause up-regulation of anti-apoptotic Bcl-2 (Graham et al., 1996), Bcl-w (Henshall et al., 2001b; Murphy et al., 2007) and Mcl-1 (Mori et al., 2004). Seizures also upregulate and activate pro-apoptotic Bax (Graham et al., 1996; Ananth et al., 2001; Henshall et al., 2002; Moran et al., 2013), trigger Bid cleavage to its truncated (most active) form (Henshall et al., 2001a; Engel et al., 2010a), induce dissociation of Bad from its cytoplasmic chaperone 14-3-3 (Henshall et al., 2002), and upregulate at a transcript and/or protein level, Bim (Shinoda et al., 2004; Murphy et al., 2010), Puma (Engel et al., 2010c), and Bmf (Moran et al., 2013).

How do Bcl-2 family proteins regulate seizure-induced neuronal death? Co-immunoprecipitation and mitochondrial enrichment analysis shows pro-apoptotic Bcl-2 family proteins functionally target anti-apoptotic members and/or cluster at mitochondria during seizure-induced neuronal death (Henshall et al., 2002; Shinoda et al., 2004; Engel et al., 2010a). This is predicted to cause release of apoptogenic proteins from mitochondria that activate downstream caspase-dependent and –independent cell death. The timing of the activation of pro-apoptotic Bcl-2 family proteins (1–4 h post status epilepticus) is broadly consistent with the release of apoptogenic molecules from mitochondria; cytochrome c and apoptosis-inducing factor are released between 1 and 4 h after status epilepticus (Henshall et al., 2000; Murphy et al., 2007; Engel et al., 2010a; Zhao et al., 2010). Nevertheless, there is no direct evidence that blocking Bcl-2 family proteins alters apoptosis signaling down-stream of mitochondria after status epilepticus.

There is also evidence that levels of Bcl-2 family members are altered in brain tissue from patients with pharmacoresistant temporal lobe epilepsy. Analysis of hippocampus and neocortex removed for the treatment of intractable seizures has found higher levels of Bcl-2, Bcl-xL, Bcl-w, and lower levels of Bim. The molecular repertoire appears shifted toward an anti-apoptotic balance which may serve to resist further neuron loss in patients experiencing frequent seizures (Henshall and Meldrum, 2012).

Several members of the Bcl-2 family have yet to be characterized in seizure models. These include anti-apoptotic Bcl-b and pro-apoptotic multi-domain Bok and BH3-only proteins Bik [the first BH3-only protein for which the mechanism of action was specifically linked to the BH3 domain (Boyd et al., 1995)], BNip3 and Noxa, among others.

## Seizures and seizure-induced neuronal death in mice lacking Bcl-2 family proteins

**Figure 2:**
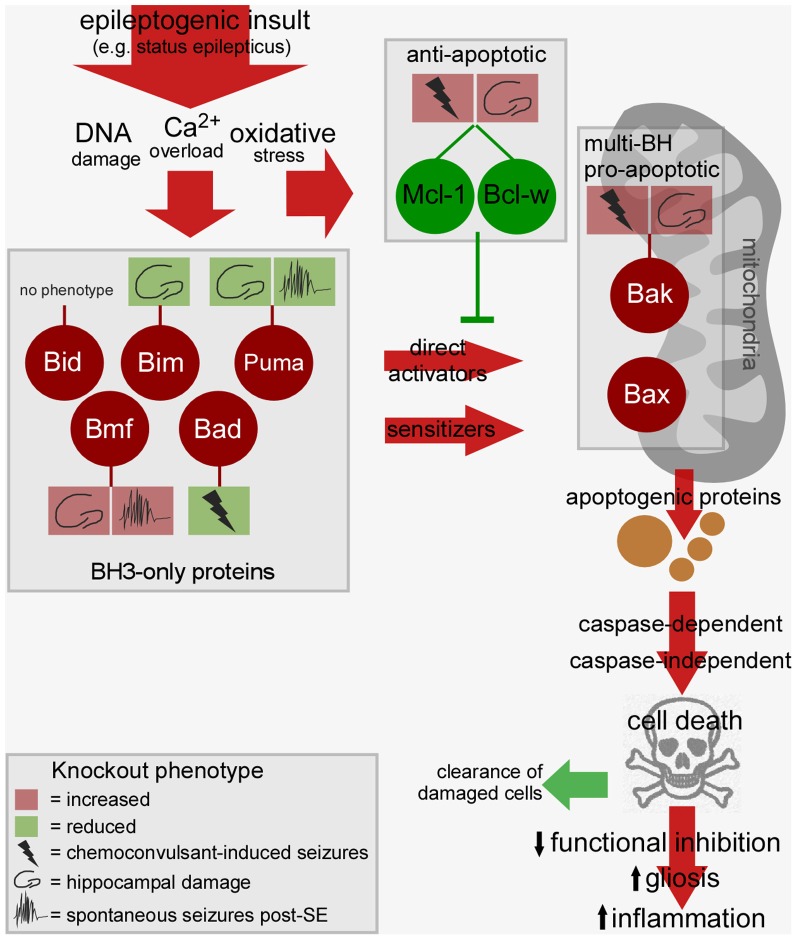
**Bcl-2 family-regulated pathway and phenotypes in Bcl-2 knockout mice after status epilepticus.** Cartoon depicts the relationship between the activated Bcl-2 family proteins after status epilepticus, the signaling pathways they drive toward cell death, and possible consequences of such cell death on epileptogenesis. Multiple BH3-only proteins are activated by epileptogenic insults which may promote mitochondrial dysfunction via Bax or other mechanisms, a step that can be blocked by anti-apoptotic members of the Bcl-2 family. The cell death controlled by this pathway may promote tissue repair or drive epileptogenesis via disruption of neuronal networks. The phenotype of Bcl-2 family protein knockout mice is indicted in the boxes linked by a stalk. For each, the key shows how deletion of the gene impacts convulsive thresholds (e.g., to kainate), hippocampal injury after status epilepticus or emergent spontaneous seizures. Not represented: The figure does not include other intracellular compartments in which Bcl-2 family proteins are commonly found such as the endoplasmic reticulum. SE, status epilepticus.

Convulsive thresholds, seizure severity and/or seizure-induced neuronal death have been assessed in knockouts of eight of the Bcl-2 family members using status epilepticus as the trigger (see Table 1). Chemoconvulsive thresholds and/or status epilepticus were found to be altered in mice lacking Bak, Mcl-1, Bcl-w and Bad, whereas loss of Bim, Bid, Puma and Bmf does not affect evoked seizures. We do not yet understand why convulsant thresholds differ in some of these mice and assessment of the neuroanatomy of these mice has in most cases ruled out any significant structural abnormalities accounting for phenotypes. Possible compensatory up-/down-regulation of other members of the Bcl-2 family have also been excluded in some, but not all, studies. For some (e.g., Bim and Bid knockout mice) assessments have been made by independent laboratories and for two members of the family, Puma and Bmf, long-term recordings have been performed to assess the impact of the loss of the gene on spontaneous (i.e., epileptic) seizures. Several members of the Bcl-2 family have not been tested as yet or their assessment remains unfeasible at present because of brain abnormalities [e.g., *Bax*^−/−^ mice, (Moran et al., 2013)]. A brief chronological review of the seizure and damage phenotypes in these mice follows and results are further summarized in Figure 2.

### Bak

Bak is predominantly expressed in neurons in the CNS (Krajewska et al., 2002) and was the first Bcl-2 family member for which mice lacking the gene were subjected to a functional assessment in status epilepticus. Contrary to expectation, mice lacking Bak displayed increased seizure-induced neuronal death following systemic kainate injection, supporting an anti-apoptotic rather than pro-apoptotic function (Fannjiang et al., 2003) (Table 1). Bak deficiency had no effect on pentylenetetrazol-induced convulsions, however, and was protective in a model of stroke (Fannjiang et al., 2003). This suggests that Bak may switch between pro- and anti-apoptotic functions according to the nature of the neurological insult. This may be possible because neurons can express a Bak splice variant that serves an anti-apoptotic function (Sun et al., 2001; Fannjiang et al., 2003). Bak deficiency was also found to result in more severe behavioral seizures (Fannjiang et al., 2003). Whether this relates to subtle differences in brain development or other effects of the loss of Bak on neuronal excitability is uncertain. It complicates, however, conclusions on whether differences in damage relate to an altered initial insult or an effect specific to signaling pathways. Verification of the impact of Bak loss in other models (e.g., pilocarpine) and direct evidence for activation *in vivo* may be warranted.

### Mcl-1

Mcl-1, first cloned in 1993 (Kozopas et al., 1993), is constitutively expressed at low levels in the adult brain, including in the main neuronal populations of the hippocampus, and Mcl-1 is strongly up-regulated in the areas that survive status epilepticus (Mori et al., 2004). A neuroprotective role for Mcl-1 was supported by the finding that heterozygous mice (*Mcl1*^+/−^) (knockout is embryonic lethal) displayed a four-fold increase in seizure-induced neuronal death following pilocarpine-induced status epilepticus (Mori et al., 2004). The study also found that heterozygotes were more sensitive to chemoconvulsant, necessitating the use of a lower dose of pilocarpine to produce comparable status epilepticus (Mori et al., 2004). As with Bak-deficient mice, this makes it somewhat difficult to conclude that effects are only due to altered cell death signaling. Nevertheless, Mcl-1 appears to be among the most important anti-apoptotic members of the family in seizure models.

### Bcl-w

Bcl-w was cloned in 1996 (Gibson et al., 1996) and is expressed in many tissues, including the brain (Hamner et al., 1999; O'Reilly et al., 2001). Protein levels of Bcl-w are bi-directionally altered in the hippocampus after seizures (Henshall et al., 2001b; Murphy et al., 2007). In damaged subfields after status epilepticus, Bcl-w appears to be targeted by Bim and becomes integrated, possibly inactivated, in the mitochondrial compartment (Shinoda et al., 2004; Murphy et al., 2007). In contrast, Bcl-w is up-regulated following brief electroshock seizures, a model of epileptic tolerance (Murphy et al., 2007). Hippocampal damage is significantly increased in Bcl-w-deficient mice following status epilepticus (Murphy et al., 2007). The mice also display a shorter time period between injection of the chemoconvulsant and their first paroxysmal seizure discharge. As a result, Bcl-w deficient mice experience a longer period of status epilepticus during monitoring (Murphy et al., 2007). A statistical analysis of the relationship between seizure duration and damage showed this could not account for all the additional damage (Murphy et al., 2007). Thus, the exacerbated hippocampal injury in Bcl-w-deficient mice is at least partly the result of losing the neuroprotective properties of the protein. As with Mcl-1 and Bak, we do not know why these knockout mice display altered responses to chemoconvulsants but it is assumed to relate to subtle effects of gene deficiency on brain development or non-cell death related functions of the proteins that impact on excitability.

### Bim

Bim was the first of the BH3-only proteins studied for a causal role in seizure-induced neuronal death. Mice lacking Bim display select abnormalities of apoptosis control (Bouillet et al., 1999) but the brain appears normal (Murphy et al., 2010). Bim is upregulated in a Foxo- or JNK-dependent manner following status epilepticus, and has been shown to co-immunoprecipitate with anti-apoptotic Bcl-2 family proteins, suggesting it is active (Shinoda et al., 2004; Murphy et al., 2010). Three studies have investigated the effect of loss of Bim, all in kainate models. In the first, kainate was directly injected into the hippocampus and in that study mice lacking Bim showed no difference in seizures or neuronal death (Theofilas et al., 2009). In contrast, a significant reduction in hippocampal damage was found in mice lacking Bim in which seizures were triggered by intra-amygdala kainate, although cortical injury was not affected (Murphy et al., 2010). Again, seizures were normal in *Bim*^−/−^ mice. The authors suggested that deficiency in Bim protects only those regions in which Bim was normally induced by seizures (Murphy et al., 2010). Other data support causal roles for Bim in excitotoxic neuronal death (Concannon et al., 2010). A third study that used systemic kainate to trigger seizures in Bim-deficient mice, in this case forebrain-specific knockouts, also found the mice underwent normal seizures (Gimenez-Cassina et al., 2012). Thus, loss of Bim has no effect on seizures although has a model- and brain region-specific contribution to seizure-induced neuronal death.

### Puma

Puma (Bbc3) is present at very low levels in the normal adult brain but undergoes rapid up-regulation after status epilepticus, driven by p53 (Engel et al., 2010c). Seizure-induced neuronal death was found to be strongly reduced in mice lacking Puma (Engel et al., 2010c). Lack of Puma also protected in a model of more severe status epilepticus, suggesting that Puma deficiency protects against necrosis (Engel et al., 2010b). The development of spontaneous seizures was also investigated in Puma-deficient and Puma-expressing mice subject to status epilepticus. Remarkably, mice lacking Puma had over 60% less epileptic seizures during the two week recordings. Also, only 63% of these mice developed any spontaneous seizures (vs. 89% of the heterozygous animals used as seizure-controls). When epileptic seizures did occur, they were of similar severity and duration (Engel et al., 2010c). The mechanism of this anti-epileptic effect is uncertain. The reduced neuronal death may have resulted in diminished gliosis and reduced inflammation. Neuroprotection may also have preserved some functional inhibition although electrophysiological studies were not performed and the type of protected neurons (e.g., glutamatergic or GABAergic) remain unidentified.

### Bid

Bid is another member of the subgroup of potently pro-apoptotic BH3-only proteins that is robustly expressed in the adult brain, including in hippocampal neurons (Krajewska et al., 2002). Previous work had shown the loss of Bid to protect against focal cerebral ischemia *in vivo* and excitotoxicity *in vitro* (Plesnila et al., 2001; Yin et al., 2002); the expectation was that Bid loss would also prevent seizure-induced neuronal death. Although Bid was activated by status epilepticus, seizure-induced neuronal death in *Bid*^−/−^ mice was similar to wild-type animals (Engel et al., 2010a). In this and another recent study, seizures were not different, indicating that Bid probably does not have a critical role in neurodevelopment or neurophysiological functions (Engel et al., 2010a; Gimenez-Cassina et al., 2012). Thus, in contrast to established pro-apoptotic roles in ischemic brain injury *in vivo*, loss of Bid does not affect seizure-induced neuronal death. This suggests the contribution of individual BH3-only proteins is dependent on the brain insult which may be because of differences in the nature of the molecular stress and how this interacts with the prevailing expression of the protein at the time of the insult (Engel et al., 2011).

### Bad

While hippocampal damage after status epilepticus in mice lacking Bad has not been reported, convulsive thresholds have been investigated. Mice lacking Bad display significantly reduced seizure severity in response to kainate and other chemoconvulsants (Gimenez-Cassina et al., 2012). No other mouse lacking a BH3-only protein has been found to have such a phenotype. The cause of the anticonvulsant effect of Bad deficiency may relate to reduced mitochondrial use of glucose and a shift to use of ketone bodies; possibly the same mechanism that underlies the anti-epileptic effect of the ketogenic diet (Danial et al., 2013) (see also below).

### Bmf

The most recently studied Bcl-2 family protein in seizure-induced neuronal death is Bmf. Transcript levels of Bmf are increased rapidly following status epilepticus in mice in a AMP kinase dependent manner, although the paucity of specific antibodies has prevented an assessment of where the protein is within the hippocampus and whether this is affected by seizures (Moran et al., 2013). Bmf-deficient mice display normal neuroanatomy and undergo normal seizures in the intra-amygdala kainate model (Moran et al., 2013). Remarkably, however, mice lacking Bmf displayed significantly more, not less, seizure-induced neuronal death than wild-types. Increased damage was mainly found in the CA3 subfield but was also evident in CA1 and the hilus and even in the contralateral hippocampus, an area normally spared in this model (Moran et al., 2013). This is the first example of a BH3-only protein apparently protecting against seizure-induced neuronal death. Nevertheless, the mechanism of this protection is unknown. Long-term EEG recordings in *Bmf*^−/−^ mice after status epilepticus found ~30% higher rate of spontaneous seizures, suggesting exacerbated hippocampal damage may worsen the form of developed epilepsy (Moran et al., 2013). Notably, the severity of individual seizures was not different in the *Bmf*^−/−^ animals supporting findings with Puma-deficient mice that modulating neuronal cell death affects the occurrence/threshold of the seizures but not the severity of the seizures once they occur (Engel et al., 2010c).

## Are there compensatory responses in knockout mice that do not display a phenotype?

It might be possible that compensatory responses in knockout mice rather than functional redundancy explain the lack of seizure phenotype in some models. This may be the case for Bid, where increased Bim is observed (Engel et al., 2010a), although this is not direct evidence of compensation. In other studies, compensatory up- or down-regulation of other Bcl-2 family proteins has not been found (Fannjiang et al., 2003; Moran et al., 2013) although the same level of a related protein may take over functions, especially in a group of proteins that are highly dependent on subcellular distribution, as is the case with the Bcl-2 family.

## Constitutive and non-canonical functions of Bcl-2 family proteins in the brain

The presence of quite high levels of several pro-apoptotic Bcl-2 family proteins in the normal adult mammalian brain is at odds with the need for neurons to survive for many years or decades; possessing high levels of proteins whose only role is to kill cells is both energy-expensive and risky. Moreover, several non-Bcl-2 family-related components of the apoptotic machinery are down-regulated in the adult brain including the apoptotic protease activating factor-1 (Apaf-1) and caspase-3 (Yakovlev et al., 2001). We now know that particular Bcl-2 family proteins have non-canonical roles, reviews of which can be found elsewhere (Lamb and Hardwick, 2010; Hardwick et al., 2012). In some cases this involves a switch to anti-apoptotic rather than pro-apoptotic function, as for Bak (Fannjiang et al., 2003) and perhaps Bmf (Moran et al., 2013). For others, however, Bcl-2 family proteins serve completely different functions in the normal brain, although remain capable of being co-opted to regulate cell death when called upon (e.g. after a severe stressor).

Bad has previously been implicated in the control of glycolysis in non-neuronal cells (Danial et al., 2003). Recent work showed that Bad is also involved in metabolism of glucose in neurons and glia (astrocytes) (Gimenez-Cassina et al., 2012). Loss of Bad resulted in a switch to ketone-based metabolism in neurons. This may phenocopy the ketogenic diet and the study reported that mice lacking Bad displayed strongly reduced seizure susceptibility to kainate and pentylenetetrazole (Gimenez-Cassina et al., 2012).

Bcl-xl contributes to mitochondrial outer membrane conductance which in turn may enhance synaptic transmission (Jonas et al., 2003; Chen et al., 2011). Bcl-xL also potentiates ATP production in neurons in the resting and active state, which appears to result from a more efficient use of oxygen in ATP production and effects on the F_1_F_0_ ATPase (Alavian et al., 2011). Whether these effects are independent of the canonical role of Bcl-xL in inhibiting apoptosis is unclear. Bcl-xL as well as several other members of the Bcl-2 family are linked to the control of intracellular calcium homeostasis (Hetz and Glimcher, 2008).

A seizure phenotype was also found in Bcl-w-deficient mice. Although the mechanism is not understood, electrophysiology and binding studies showed that Bcl-w can augment GABA-evoked currents in neurons (Murphy et al., 2007).

These emerging, non-cannonical functions of Bcl-2 family proteins in the brain are an exciting focus of research that may yield mechanisms relevant to epileptogenesis independent of their traditional roles in controlling cell death. Modulating certain Bcl-2 family proteins that have direct effects on excitability is an alternative tactic that may complement the neuroprotective therapeutic opportunities of targeting this gene family.

## Summary and future directions

Neuronal loss is a potential consequence of prolonged or repeated brief seizures and may contribute to epileptogenesis. The availability of Bcl-2 family knockout mice has enabled epilepsy researchers to learn about the functional contributions of this gene family to seizure-induced neuronal death. Eight members of this family have been assessed *in vivo* using genetic models, representing one of the most comprehensive analyses of any single gene family in the epilepsy field. The studies have significantly expanded our understanding of the influence of apoptosis-associated signaling pathways in epileptogenesis, revealing effects on convulsant thresholds and neuronal death. For two members, altered spontaneous seizures were also reported in mice lacking a Bcl-2 family member supporting neuronal death controlled by these genes as having direct or indirect effects on functional inhibition in the brain.

What are some of the next challenges? Studies are needed to test network function and explore the underlying direct and indirect mechanisms by which modulation of Bcl-2 family proteins influences cell death and excitability. Bcl-2 family proteins have important non-cell death functions in neurons, some of which are directly relevant to the excitability of the brain and thus may be novel treatment targets in epilepsy. We know relatively little about which hippocampal cell type(s) make these proteins. There is an assumption that all effects are neuronally-mediated but this needs to be established. All work to date has used knockouts and there is a need to investigate whether transgenic overexpression of an anti-apoptotic Bcl-2 family protein such as Mcl-1 can reduce seizure damage and/or have an anti-epileptogenic effect. There remain important members of the family for which we have little or no data on their influence on seizures and damage in a genetic model *in vivo*, including Bcl-xL. The possibility that some Bcl-2 family proteins can compensate for others could be tested using double (or even triple) knockout mice, which are now available (Ren et al., 2010). This may be particularly apt for Bid where the absence of a damage phenotype in the knockout was unexpected. Another key experiment is to prove that seizure-induced cell death is really Bax-dependent *in vivo*. Bax-deficient mice display brain abnormalities that are presumably a result of deficiencies in developmental cell death (Moran et al., 2013), necessitating the use of conditional Bax knockout mice. Conditional knockouts or transgenic mice would also provide a means to test whether the pro/anti-convulsive seizure thresholds in certain Bcl-2 family knockouts (see Table 1) are simply an effect of the loss of the gene during brain development or instead represent an effect relating to neurophysiologic functions of the protein in the adult brain. Finally, can the knowledge be applied therapeutically? We know the transcriptional and post-transcriptional control mechanisms for many of the Bcl-2 family proteins and these provide potential drug targets. The availability of BH3 mimetics, developed for use in cancer, offer tools to probe the contribution of Bcl-2 family proteins in seizure models but we await small molecules that enhance or mimic the actions of anti-apoptotic members of the Bcl-2 family that might be put to use to protect the brain against injury caused by status epilepticus and, perhaps, epileptogenesis.

### Conflict of interest statement

The authors declare that the research was conducted in the absence of any commercial or financial relationships that could be construed as a potential conflict of interest.
